# Glutamatergic Fate of Neural Progenitor Cells of Rats with Inherited Audiogenic Epilepsy

**DOI:** 10.3390/brainsci10050311

**Published:** 2020-05-21

**Authors:** Alexandra A. Naumova, Ekaterina A. Oleynik, Elena V. Chernigovskaya, Margarita V. Glazova

**Affiliations:** Sechenov Institute of Evolutionary Physiology and Biochemistry of the Russian Academy of Sciences, 44 Thorez pr., 194223 St. Petersburg, Russia; alexandra.naumova@iephb.ru (A.A.N.); ekaterina.oleynik@iephb.ru (E.A.O.); elena.chernigovskaya@iephb.ru (E.V.C.)

**Keywords:** Krushinsky-Molodkina rats, hippocampus, neuronal differentiation, PKA, ERK1/2, Akt, GSK3β

## Abstract

Epilepsy is associated with aberrant neurogenesis in the hippocampus and may underlie the development of hereditary epilepsy. In the present study, we analyzed the differentiation fate of neural progenitor cells (NPC), which were isolated from the hippocampus of embryos of Krushinsky-Molodkina (KM) rats genetically prone to audiogenic epilepsy. NPCs from embryos of Wistar rats were used as the control. We found principal differences between Wistar and KM NPC in unstimulated controls: Wistar NPC culture contained both gamma-aminobutyric acid (GABA) and glutamatergic neurons; KM NPC culture was mainly represented by glutamatergic cells. The stimulation of glutamatergic differentiation of Wistar NPC resulted in a significant increase in glutamatergic cell number that was accompanied by the activation of protein kinase A. The stimulation of KM NPC led to a decrease in immature glutamatergic cell number and was associated with the activation of extracellular signal-regulated kinases 1 and 2 (ERK1/2) and protein kinase B/ glycogen synthase kinase 3 beta (Akt/GSK3β), which indicates the activation of glutamatergic cell maturation. These results suggest genetically programmed abnormalities in KM rats that determine the glutamatergic fate of NPC and contribute to the development of audiogenic epilepsy.

## 1. Introduction

Audiogenic epilepsy is a form of reflex epilepsy and its etiology is mainly hereditary, both in human and animal [1,2,3]. The development of audiogenic epilepsy, along with other epilepsy types, involves many factors, including aberrant neuronal excitability and a disturbed balance between inhibitory and excitatory synaptic transmission in the brain [4]. At the same time, it is known that epileptogenesis is associated with aberrant neurogenesis in the hippocampus [5,6].

Observations of patients with epilepsy and the results of experimental modeling of seizures indicate that epileptiform activity stimulates proliferation of neural progenitors in the subgranular layer of the dentate gyrus [7,8,9,10]. Part of these newborn cells migrates to the hilus and differentiates into ectopic excitatory glutamatergic neurons that exhibit stable hyperactivity and tend to synchronize with Cornu Ammonis area 3 (CA3) pyramid cells of the hippocampus [10,11]. On the other hand, the inhibition of aberrant neurogenesis caused by status epilepticus significantly reduced the frequency and severity of seizures [12,13].

Nowadays, genetic abnormalities in epilepsy are being actively investigated. Mutations in a variety of genes encoding voltage-dependent ion channels, receptors, synaptic proteins were identified to underlie human congenital epileptic syndromes [14,15]. Interestingly, some of these genes also participate in the regulation of neurogenesis [16,17,18,19]. These data led us to hypothesize that aberrant neurogenesis can be not only a consequence of epileptiform activity, but also a cause, in the case of hereditary epilepsy.

Key roles in the regulation of neurogenesis during development and in the adult brain belong to extracellular signal-regulated kinases 1 and 2 (ERK1/2), protein kinase A (PKA) and protein kinase B (Akt), as well as to glycogen synthase kinase 3 beta (GSK3β). GSK3β is the component of canonic Wnt signaling pathway that regulates differentiation and morphogenesis of maturing neurons [20,21,22,23]. Its activity is modulated by multiple signaling including PKA and Akt [24]. In particular, both PKA and Akt can regulate GSK3β by phosphorylation at Ser9. On the other hand, the activation of ERK1/2, PKA, and Akt was reported in the hippocampus, cortex, and in neural stem cells of the dentate gyrus after the expression of seizures [25,26,27,28], and in human epileptic brain [29,30,31]. These alterations can be associated with increased neurogenesis in epileptic brain.

In the present study, we used the inbred Krushinsky-Molodkina (KM) rats, which were selected from Wistar rats [32]. KM rats are genetically prone to audiogenic seizures and demonstrate stable sound-induced tonic-clonic seizures in an age-dependent manner. The increased convulsive readiness of these rats is fully established by the end of the third month of postnatal development [32]. At the same time, genetic abnormalities, including congenital defects of neurogenesis in KM rats, are poorly understood. Here, we analyzed the differentiation fate of neural progenitor cells (NPC) of KM rats isolated from the hippocampi of the embryos and compared it with Wistar NPC behavior. We determined dramatic differences between KM and Wistar NPC even at baseline condition when cells were incubated without growth factors. In control (unstimulated) culture KM, NPC were mostly presented as immature glutamatergic cells, while in unstimulated Wistar NPC culture both gamma-aminobutyric acid (GABA) and glutamatergic cell types were combined. After stimulation of glutamatergic differentiation, we observed the activation of ERK1/2 and Akt signaling in KM NPC, while in Wistar NPC we revealed activation of PKA. Thus, our data demonstrated the differences in the mechanisms, which mediate NPC differentiation between Wistar and KM rats. Taken together, the glutamatergic fate of KM NPC and differences in the mechanisms of differentiation between Wistar and KM NPC suggested that genetically determined aberrations of neurogenesis are involved in epileptogenesis.

## 2. Materials and Methods

### 2.1. Animals

Pregnant female KM and Wistar rats, at 18–19 gestation days, were recruited in the experiments. The rats were housed in individual cages under natural light–dark cycle with free access to food and water. Before isolation of embryos, the rats were euthanized with CO2. All procedures were approved by the Institutional Animal Care and Use Committee at the Sechenov Institute of Evolutionary Physiology and Biochemistry (#2/2019, approved on 20.02.2019).

### 2.2. Isolation of Neural Progenitor Cells (NPC)

All manipulations were performed in sterile conditions. Rat embryos were isolated and decapitated; hippocampal areas were dissected and mechanically dissociated in cold (4 °C) Hank’s solution (Biolot, St. Petersburg, Russia; #1.2.1.6). In order to further separate the cells, tissue homogenates were incubated in 0.25% trypsin/EDTA medium at 37 °C for 30 min. Then trypsin was inhibited by 20% fetal bovine serum (ThermoFisher Scientific, Gibco™, Waltham, Massachusetts, USA; #10082139) and suspensions were centrifuged at 1000 rpm for 5 min. Cell pellets were resuspended in DMEM (ThermoFisher Scientific, Gibco™, #41966029) with 10% inactivated horse serum (ThermoFisher Scientific, Gibco™, #26050-088), 5% fetal bovine serum, and penicillin/streptomycin (Biolot, #1.3.18) and incubated in cell-culture-treated Petri dishes for neurospheres formation. After 3–4 days, the neurospheres were mechanically dissociated and seeded in 6-well cell culture-treated plates. One sterile coverslip (12 mm diameter) was placed in each well to have the same cells for immunocytochemical and Western blot analysis. The cells were incubated in Neurobasal medium (ThermoFisher Scientific, Gibco™, #21103049) with L-glutamine (Biolot, #1.3.8.2), B27 supplement (ThermoFisher Scientific, Gibco™, #17504044), EGF (epidermal growth factor, 20 ng/mL, ThermoFisher Scientific, Gibco™, #PMG8041), FGFb (basic fibroblast growth factor, 20 ng/mL, PeproTech, Rocky Hill, NJ, USA.; #450-33), and heparin (50 ng/mL, Merck, Sigma-Aldrich, St. Louis, MO, USA; #H0200000) until monolayers were formed. Heparin was added to maintain FGFb activity [33].

### 2.3. Glutamatergic Differentiation Protocol

To stimulate glutamatergic differentiation, in 3–4 days Neurobasal/B27/EGF/bFGF medium was changed for Neurobasal/B27 with BDNF (brain-derived neurotrophic factor, 20 ng/mL; PeproTech, Rocky Hill, NJ, USA; #450-02), GDNF (glial cell-line-derived neurotrophic factor, 10 ng/mL, PeproTech, #450-51), IGF1 (insulin-like growth factor 1, 40 ng/mL, PeproTech, #250-19), and FGF7/KGF (fibroblast growth factor 7/keratinocyte growth factor, 40 ng/mL, PeproTech, #450-60). Monolayer cultures of NPC were incubated for 5 days. Control groups of Wistar and KM NPC were incubated for 5 days in Neurobasal/B27 without growth factors. After incubation, the coverslips with growing cells were fixed in 4% formalin for immunofluorescent assay, the rest of cells were harvested in lysis buffer for Western blot analysis.

### 2.4. Immunofluorescent Staining

Double immunofluorescent staining was made to reveal the co-expression of vesicular glutamate transporter 1 and 2 (VGLUT1/2) or glutamate decarboxylase 65 and 67 (GAD65/67), with doublecortine (DCX) in NPC of control and stimulated groups. After standard preliminary processing, NPC-containing coverslips were incubated with primary antibodies against GAD65 (1:250, Abcam, Cambridge, UK; #ab26113) and GAD67 (1:300, Merck, Darmstadt, Germany; #MAB5406) or VGLUT1 (1:200, Merck, Sigma-Aldrich, #MAB5502) and VGLUT2 (1:500, Merck, Sigma-Aldrich, #MAB5504) in combination with primary antibodies against DCX (1:300, Cell Signaling Technology, Danvers, MA, USA; #4604). Primary antibodies were visualized by anti-rabbit AlexaFluor 488 (1:1000; Invitrogen, Carlsbad, CA, USA; #913909) and anti-mouse AlexaFluor 568 (1:1000, Invitrogen, #762708). Cell nuclei were stained by 4’,6-diamino-2-phenylindole (DAPI) (Merck, Sigma-Aldrich, #D9542).

### 2.5. Western Blotting

NPC cultures were harvested in hot (95 °C) SDS buffer (4% SDS, 0.13 M Tris-HCl, pH 6.7, 10% glycerol, 0.002% bromphenolblue, 10% β-mercaptoethanol; 100 µl per well), incubated at 95 °C for 10 min, and stored at −20 °C. Equal amounts of samples (5 µl per line) were loaded for electrophoresis and proteins were separated on 10% polyacrylamide gel and then transferred to a nitrocellulose membrane (Santa Cruz, Dallas, TX, USA; #sc-3718). The membranes were incubated in 3% non-fat milk or 3% BSA in TBST buffer (0.1% Tween 20, 20 mM Tris, 137 mM NaCl, pH 7.4) for 1 h and then incubated overnight at 4°C with primary antibodies. Used antibodies: ERK1/2 (1:1000; Cell Signaling Technology, #9102), phospho-ERK1/2 (Thr202/Thr204, 1:1000; Cell Signaling Technology, #4376), Akt (1:1000, Cell Signaling Technology, #4691), phospho-Akt (Thr308, 1:1000, Cell Signaling Technology, #2965), GSK3β (1:1000, Cell Signaling Technology, #9315), phospho-GSK3β (Ser9, 1:1000, Cell Signaling Technology, #5558), α/β-tubulin (1:2000, Cell Signaling Technology, #2148), and phosphorylated substrates of PKA (1:1000, Cell Signaling Technology, #9624). Then, the membranes were washed in TBST buffer and incubated with HRP-conjugated secondary anti-rabbit antibodies (1:10,000, Vector Laboratories, Burlingame, CA, USA; #PI-2000) for 1 h at room temperature. After extensive washing, the membranes were incubated for 5 min in SuperSignal™ West Dura Extended Duration Substrate (ThermoFisher Scientific, #34075) to produce chemiluminescent reaction.

### 2.6. Evaluation of Cell Cultures and Statistical Analysis

Cell cultures were analyzed by counting the numbers of cells immunopositive for specific markers GAD65/67, VGLUT1/2, and DCX in each group. The total cell numbers in the groups were determined by counting DAPI-stained cell nuclei. Digital pictures were obtained with use of Leica DMI 6000B fluorescent microscope (Leica Microsystems GmbH, Wetzlar, Germany) and then processed by ImageJ software for the cell counting.

For Western blot assay, densitometric analysis of protein content was performed using ImageJ. Phosphorylation of ERK1/2, Akt, and GSK3β was estimated as ratios pERK1/2/ERK1/2, pAkt/Akt and pGSK3β/GSK3β. Phosphorylation of PKA substrates was estimated by normalizing to tubulin.

All data were processed statistically by the Mann–Whitney U test with use of GraphPad Prism 8 software. The results are presented as mean ± SD. Differences were regarded as significant at *p* < 0.05.

## 3. Results

### 3.1. Analysis of Differentiation Fate

**Figure 1:**
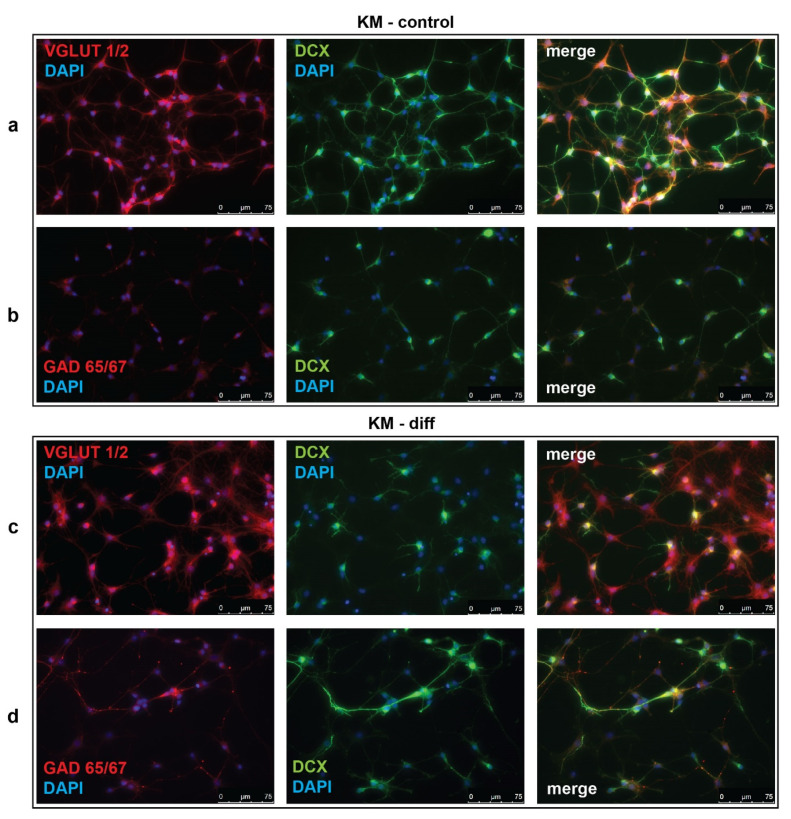
Immunofluorescent analysis of vesicular glutamate transporter 1 and 2 (VGLUT1/2), glutamate decarboxylase 65 and 67 (GAD65/67), and doublecortin (DCX) in cultured neural progenitor cells (NPC) isolated from Krushinsky–Molodkina (KM) rat embryos. (**a**,**c**) NPC of KM rats were stained for VGLUT1/2 (red) or (**b**,**d**) GAD65/67 (red) in combination with DCX (green) in control (KM-control) and neurotrophin-stimulated cultures (KM-diff). Cell nuclei were stained by 4’,6-diamino-2-phenylindole (DAPI) (blue). Merged images demonstrate cells with co-localization of VGLUT1/2 or GAD65/67 with DCX. Data are representative of three independent experiments.

**Figure 2:**
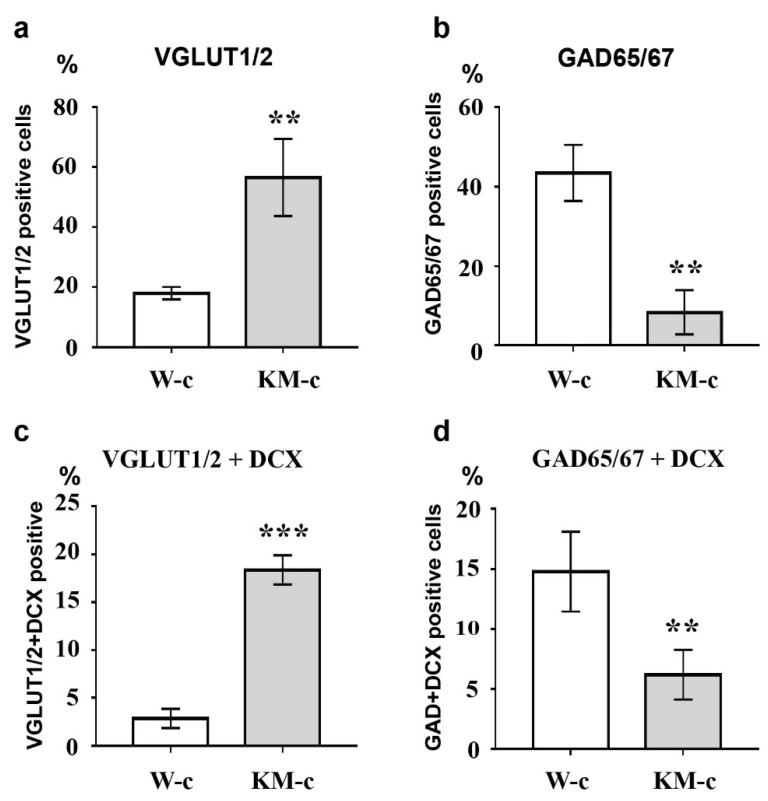
Analysis of VGLUT1/2, GAD65/67, and doublecortin (DCX) expression in control cultures of NPC. Control culture of KM NPC (KM-c) demonstrated increased numbers of VGLUT1/2- (**a**), and VGLUT1/2/DCX-double positive (**c**) cells in comparison with control Wistar NPC culture (W-c). In contrast, percentages of GAD65/67-positive (**b**) and GAD65/67/DCX-double positive (**d**) cells were higher in control Wistar culture. *Axis x*: experimental groups. *Axis y*: number of immunopositive or double immunopositive cells as % of the whole cell number calculated with use of DAPI-stained cell nuclei. The data are calculated from three independent experiments. Results are shown as mean ± SD. Significant differences from control: ** *p* < 0.005, *** *p* < 0.001.

**Figure 3:**
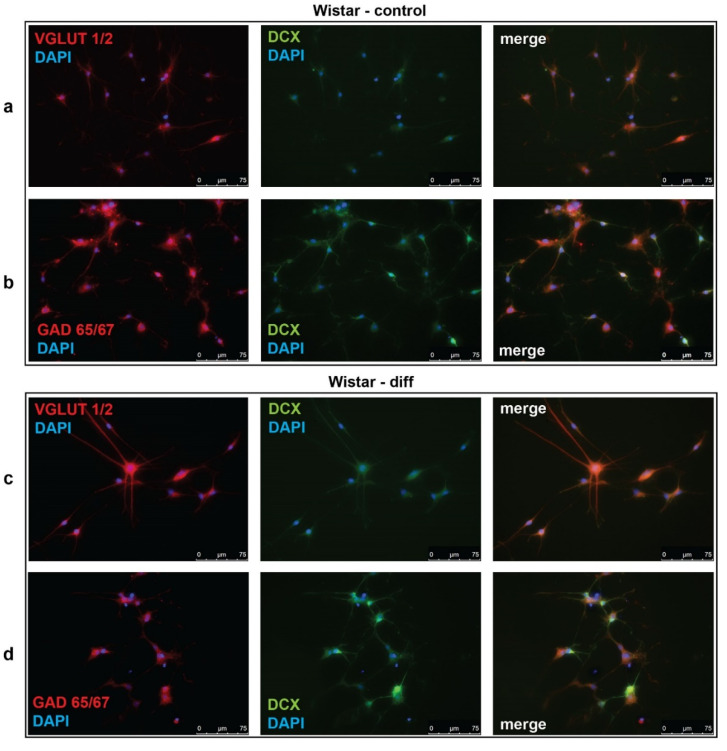
Representative micrographs of cultured neural progenitor cells (NPC) isolated from Wistar rat embryos. Immunofluorescent detection of VGLUT1/2, GAD65/67, and doublecortin (DCX). (**a**,**c**) NPC of Wistar rats were stained for VGLUT1/2 (red) or (**b**,**d)** GAD65/67 (red) with DCX (green) in baseline conditions (Wistar-contol) and after neurotrophin-stimulated glutamatergic differentiation (Wistar-diff). Cell nuclei were stained by DAPI (blue). Merged images demonstrate cells with co-localization of VGLUT1/2 (**a**,**c**) or GAD65/67 (**b**,**d**) with DCX. Data are representative of three independent experiments.

To investigate the effectiveness of glutamatergic differentiation protocol, we carried out immunofluorescent detection of vesicular glutamate transporters 1 and 2 (VGLUT1/2) and glutamate decarboxylases 65 and 67 (GAD65/67), which are markers of glutamatergic and GABAergic neurons, respectively. Percentages of VGLUT1/2- and GAD65/67-positive cells were evaluated in control (unstimulated) and neurotrophin-stimulated cultures of NPC isolated from Wistar and KM rat embryos. The obtained data revealed significant difference between control groups of KM and Wistar NPC. We showed that in culture of KM NPC, the number of VGLUT1/2-positive cells was significantly higher (Figure 1a, Figure 2a), while the number of GAD65/67-positive cells was decreased (Figure 1b, Figure 2b) in comparison with Wistar NPC culture (Figure 2b, Figure 3b).

We also analyzed the expression of doublecortin (DCX), a marker of immature migrating neurons in the developing and adult brain [34]. Our data demonstrated that, in control KM NPC culture, the percentage of progenitor DCX-positive cells was increased in comparison with Wistar NPC culture (Figure 1a,b, Figure 2c, Figure 3a,b). Moreover, the expression of DCX was observed only in one-third of VGLUT1/2 cells, indicating that most of glutamatergic neurons in KM NPC culture were completely differentiated (Figure 1a, Figure 2d). Analysis of DCX expression in GAD65/67-positive cells showed that in control KM NPC culture almost all cells were double-positive (Figure 1b, Figure 2e) in contrast to Wistar NPC culture, where GABAergic cells were matured in the majority (Figure 2e, Figure 3b). These data indicated that GABAergic differentiation was more active in Wistar NPC culture, while KM NPC mainly differentiated into glutamatergic neurons.

**Figure 4:**
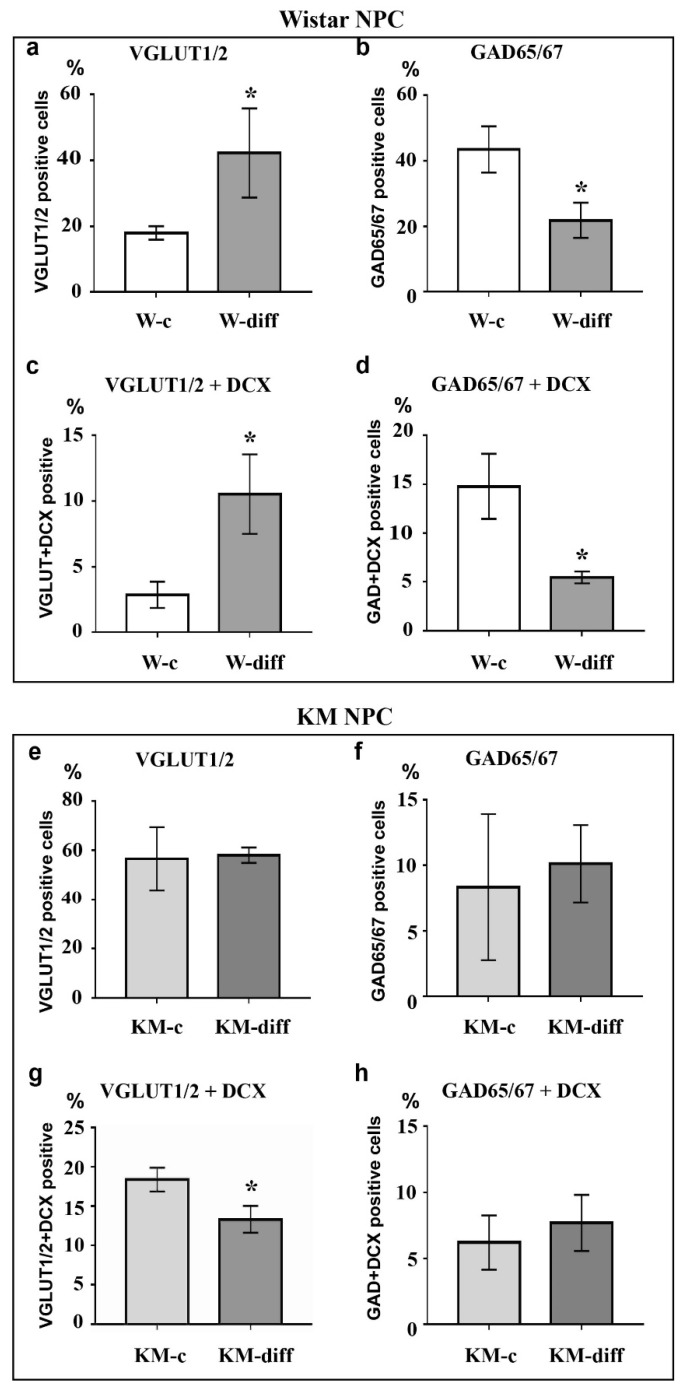
Analysis of VGLUT1/2, GAD65/67, and doublecortin (DCX) expression in cultured NPC after stimulation of glutamatergic differentiation. Stimulation of Wistar NPC culture (W-diff) induced the significant increase in the number of VGLUT1/2-positive (**a**) and VGLUT1/2/DCX-double positive (**c**) cells and decrease in the number of GAD65/67-positive (**b**) and GAD65/67/DCX-double positive (**d**) cells as compared with control (W-c). The same treatment of KM NPC (KM-diff) did not evoke any considerable changes in VGLUT1/2 (**e**) and GAD65/67 (**f**) expression as well as in co-expression of GAD65/67 (**h**) with DCX in comparison to control (KM-c). However, the number of VGLUT1/2/DCX double positive cells in KM NPC culture was decreased after stimulation (**g**). *Axis x:* experimental groups. *Axis y:* number of immunopositive or double immunopositive cells as % of the whole cell number calculated with use of DAPI-stained cell nuclei. The data are calculated from three independent experiments. Results are shown as mean ± SD. Significant differences from control: * *p* < 0.05.

Analysis of Wistar NPC cultures after stimulation of glutamatergic differentiation by special combination of the neurotrophins revealed a dramatic increase in VGLUT1/2-positive cell number (Figure 3c, Figure 4a) in comparison with corresponding Wistar control (Figure 3a, Figure 4a), and about 25% of VGLUT1/2-positive cells expressed DCX (Figure 3c, Figure 4c). Simultaneously, the percentage of GAD65/67-positive cells was reduced (Figure 2b,d, Figure 4b,d). These data suggested that our differentiation protocol was successful to promote glutamatergic differentiation in wild-type NPC. However, the same stimulation of KM NPC towards glutamatergic differentiation changed neither glutamatergic (Figure 1a,c, Figure 4e) nor GABAergic cell percentages in the culture (Figure 1b,d, Figure 4f,i), but the percentage of DCX/VGLUT1/2 double-positive cells was significantly decreased, which suggested the activation of glutamatergic maturation (Figure 1a,c, Figure 4g).

### 3.2. Analysis of Cell Signaling

Then we analyzed activity of protein kinases ERK1/2, Akt, PKA, and GSK3β that participate in the regulation of neuronal differentiation.

**Figure 5:**
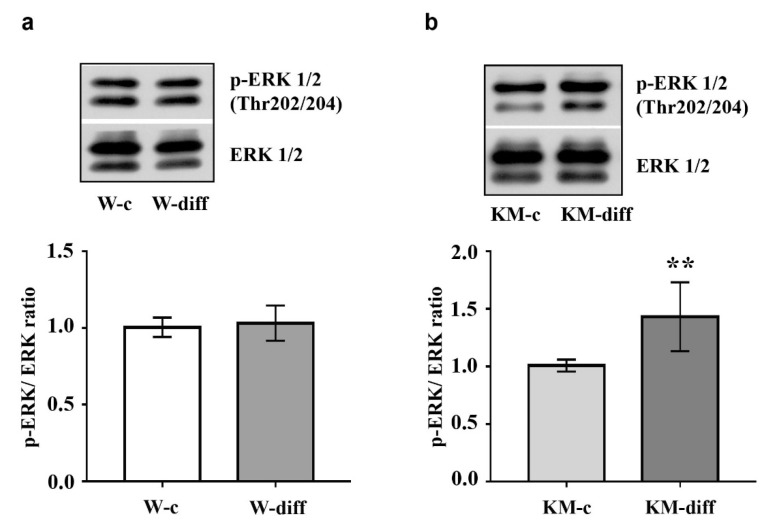
Evaluation of extracellular signal-regulated kinase 1 and 2 (ERK1/2) phosphorylation in cultured embryonic NPC of Wistar and KM rats. (**a**) Western blot analysis revealed no difference in expression of phosphorylated ERK1/2 (p-ERK1/2) in Wistar NPC of control group (W-c) and after stimulation of glutamatergic differentiation (W-diff). (**b**) Glutamatergic differentiation of KM NPC (KM-diff) was accompanied with significant increase of p-ERK1/2 as compared with unstimulated KM NPC (KM-c). Western blot data are calculated from three independent experiments. Results are presented in arbitrary units and expressed as mean ± SD. ** *p* < 0.005.

**Figure 6:**
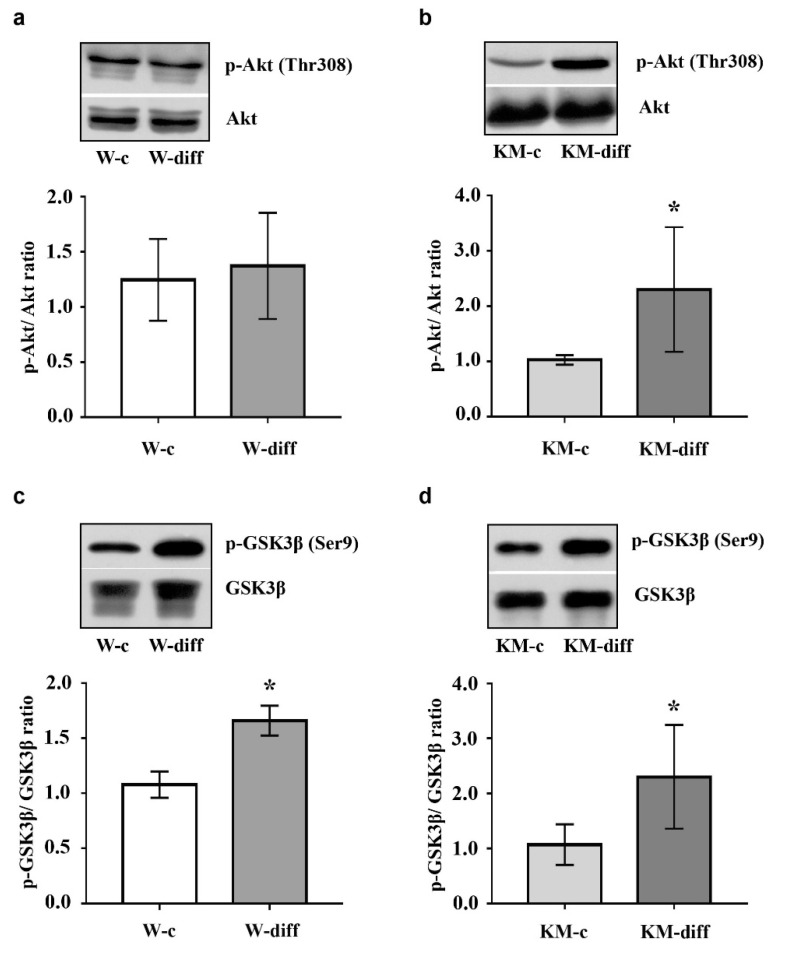
Western blot analysis of protein kinase B (Akt) and glycogen synthase kinase 3 beta (GSK3β) phosphorylation in KM and Wistar NPC. (**a**,**b**) Expression of phosphorylated Akt (p-Akt) in cultured NPC of Wistar (**a**) and KM (**b**) rat embryos in control groups (W-c, KM-c) and after stimulated glutamatergic differentiation (W-diff, KM-diff). (**c**,**d**) p-GSK3β expression in stimulated Wistar (**c**) and KM (**d**) NPC after glutamatergic differentiation (W-diff, KM-diff) as compared with corresponding controls (W-c, KM-c). Expression of p-Akt demonstrated significant increase only in stimulated KM NPC culture, while p-GSK3β was elevated in both cell cultures after differentiation. Data are calculated from three independent experiments. Results are presented as mean ± SD. * *p* < 0.05.

**Figure 7:**
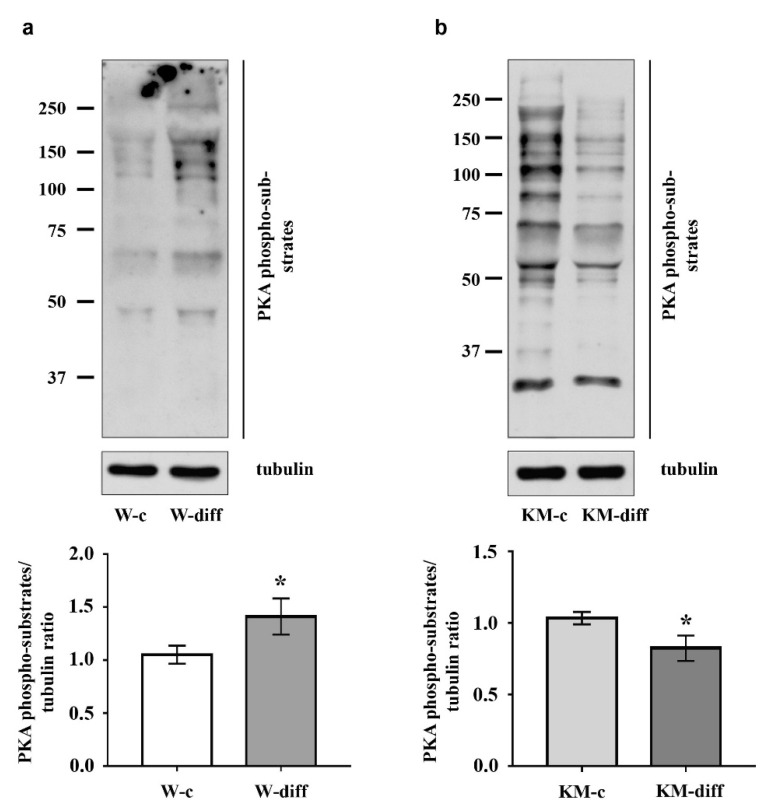
Evaluation of phosphorylated substrates of protein kinase A (PKA) in cultured NPC of Wistar and KM rat embryos. (**a**) Western blot analysis demonstrated increased phosphorylation of overall PKA substrates (PKA phospho-substrates) in Wistar NPC stimulated for glutamatergic differentiation (W-diff) as compared with corresponding control (W-c). (**b**) In KM NPC glutamatergic differentiation (KM-diff) was associated with lower content of phosphorylated substrates of PKA. Western blot data are calculated from three independent experiments. Results are shown as mean ± SD. * *p* < 0.05.

The data showed that stimulation of glutamatergic differentiation in Wistar NPC did not change the activity of ERK1/2 (Figure 5a) and Akt (Figure 6a) as compared with unstimulated Wistar NPC. At the same time, the activity of PKA was significantly enhanced (Figure 7a) that was accompanied with an increase in GSK3β phosphorylation at Ser9 (Figure 6c).

However, in stimulated KM NPC we observed significantly increased activity of ERK1/2 (Figure 5b) and Akt (Figure 6b). Phosphorylation of GSK3β was also increased (Figure 6d), while the activity of PKA was diminished in comparison with corresponding KM control (Figure 7b). These data suggest that in Wistar, the NPC activity of GSK3β can be regulated by PKA, while in KM NPC it is mainly dependent on Akt.

## 4. Discussion

An imbalance between excitatory and inhibitory signals in the brain with excessive activity of excitatory glutamatergic system is one of the basic mechanisms of seizure susceptibility [35,36]. Our recent studies demonstrated increased glutamatergic transmission in the hippocampus of KM rats during the first month of postnatal development when the readiness to audiogenic seizures has not manifested yet [37]. These results indicate that the hyperactivity of glutamatergic system may be a result of aberrant glutamatergic differentiation of new-born cells in the hippocampus of KM rats. Indeed, here we revealed predominant glutamatergic differentiation of KM NPC. Comparison of NPC in control groups, where the cells were cultured in neurotrophin-free conditions, showed dramatic differences between Wistar and KM NPC behavior. While Wistar NPC culture contained mixed population of GABA and glutamatergic cells, KM NPC culture was represented mostly by glutamatergic cells. Thus, we revealed the susceptibility of KM NPC to differentiation into excitatory glutamatergic neurons along with the impaired formation of inhibitory GABAergic neurons. These results confirm that recent data were obtained with cultures of NPC isolated from hippocampus of KM rats at the early stage of postnatal development [38]. In particular, it was shown that stimulation by the retinoic acid of KM NPC, but not Wistar NPC, led to predominant differentiation into excitatory glutamate- and dopaminergic neurons [38].

In addition, we observed that control culture of KM NPC contained a large population of immature DCX-positive neurons, which mostly were glutamatergic. In previous studies, we demonstrated an increase in VGLUT2 expression in the dentate gyrus of two-week old KM rats. However, in the dentate gyrus of one-month old KM rats expression of VGLUT2 was the same with Wistar rats [37]. Development of the dentate gyrus in rodents continues during two first weeks after birth and at the beginning is characterized by excessive formation of cells, which are then eliminated [39]. The increase in VGLUT2 expression in the dentate gyrus of two-week old KM demonstrated aberrantly activate glutamatergic transmission [37] that revealed the predisposition of newly born cells to differentiate into glutamatergic neurons. Here, we confirmed our in vivo data and demonstrated genetically determined glutamatergic differentiation fate of embryonic KM NPC in vitro. Previously, we have shown an increase in the expression of the NR2B subunit of N-methyl D-aspartate (NMDA) receptors in the hippocampus of two-week old KM pups [37]. It is known that not only neurons, but also astrocytes, both express glutamate receptors including NR2B [40] by which astrocytes synchronize Ca2+ signaling with neurons after the onset of epileptiform activity [41]. On the other hand, the astrocytes, like neurons, release glutamate and thus regulate the activity of neuronal NMDA receptors [42]. However, the inhibition of NMDA receptors [42,43,44] or disruption of astrocyte synchronization [41] reduces the severity of seizures. However, there are no data of the role of tripartite glutaminergic synapses in genetic epilepsy and this question should be address to further studies.

Then, we analyzed how the specific stimulation of glutamatergic differentiation can change NPC behavior. The stimulation of Wistar NPC led to a significant increase in glutamatergic cell number and a decrease in NPC differentiation into GABAergic cells, proving the specificity of the experimental protocol. Surprisingly, the same protocol failed to induce any significant changes in KM NPC culture, where the number of glutamatergic cells remained abnormally high.

To study the mechanisms underlying glutamatergic differentiation, we analyzed the activity of several key kinases, which regulate neuronal differentiation, such as ERK1/2, GSK3β, Akt and PKA. The first one was ERK1/2 kinase, the crucial participant of the ERK1/2 signaling cascade involved in neuronal differentiation [45] and the regulation of NMDA receptor expression [46]. The activation of ERK1/2 in the hippocampus was also demonstrated after seizure expression, stimulated by a different typed of convulsants [47,48], while the inhibition of ERK1/2 successfully prevented audiogenic seizure expression in KM rats [49]. Moreover, the hyperactivity of ERK1/2-dependent signaling cascade contributes to seizure expression [44,50]. It was shown that the activation of ERK1/2 in the hippocampus provoked spontaneous seizures in mice [44,50]. In human, high and persistence activity of ERK1/2 was revealed in the ‘epileptic’ cortex of patients with neocortical epilepsy [31]. The participation of Akt and PKA was also revealed in the patients with pharmacoresistant epilepsy. Recently, Valmiki and co-authors demonstrated sustained active Akt in the hippocampus of patients with temporal lobe epilepsy [29]. A role of PKA signaling was also confirmed in the development of pharmacoresistant temporal lobe epilepsy [30]. We determined that the activity of ERK1/2 was not changed after the differentiation of Wistar NPC into glutamatergic cells. At the same time, glutamatergic differentiation of KM NPC was accompanied with s significant elevation in ERK1/2 activity. Interestingly, during the early postnatal development of KM rats, the delayed development of the hippocampus was associated not only with the high activity of ERK1/2, but also with increased NR2B expression [32]. The present in vitro data showed an unchanged number of glutamatergic cells after stimulation that was accompanied with a significant decrease in immature glutamatergic cells and activated ERK1/2. Together, these data indicate the stimulation of glutamatergic maturation.

Protein kinase GSK3β also plays an important role in the regulation of neurogenesis during normal development and under pathological conditions [51,52]. It is well-known that GSK3β phosphorylates and inhibits several transcription factors, such as c-Myc, c-jun, β-catenine, which stimulate the proliferation of neural stem cells, therefore GSK3β activation induces neuronal differentiation [51]. The basic regulatory mechanisms that switch GSK3β activity in the brain include dynamical phosphorylation/dephosphorylation at Ser9 of GSK3β N-terminal domain. Thus, a number of neurotrophins stimulate Akt that, in turn, negatively regulates GSK3β by phosphorylation at Ser9 supporting stem cell survival or keeping the proliferative status of progenitor cells [53]. The same regulatory site of GSK3β is the target for PKA phosphorylation [24]. PKA-induced GSK3β inhibition is also supposed to be associated with cell survival and prevention of apoptosis during neurogenesis [54]. At the same time, it was shown that PKA activation stimulates neuronal differentiation in cultured cells [55,56]. Our data demonstrated that the stimulation of glutamatergic differentiation resulted in the increase in GSK3β phosphorylation at Ser9 in both Wistar and KM NPC. But in KM NPC we observed the activation of Akt, while in Wistar NPC, the activation of PKA was revealed. These data suggest that, in the case of Wistar NPC stimulation with neurotrophins, the activation of PKA is induced, which, in turn, phosphorylates multiple intracellular substrates including GSK3β and results in the expected glutamatergic differentiation. In KM NPC, the same stimulation resulted in the activation of ERK1/2 and Akt/GSK3β, signaling maintained aberrant glutamatergic differentiation.

Thus, our data suggest the genetically determined differentiation of NPC into glutamatergic neurons in the hippocampus of KM rats. We suppose that these defects contribute to the abnormal formation of the hippocampal glutamatergic system and, therefore, are responsible for the development of audiogenic seizure susceptibility in KM rats. Several studies have demonstrated that the PKA, Akt and ERK1/2 signaling pathways contribute to the development of human epilepsy too [29,30,31], which suggests that our findings may make some contribution to understanding the mechanisms of human epilepsy. However, it is important to note that our findings are based on 2D culture of NPC. The primary cultures of hippocampal cells, which contain both neurons and glia cells, or 3D perfused cellular models, can provide a more physiologically relevant insight into the mechanisms underlying signal transduction in hereditary reflex epilepsy.
